# Feasibility of home-based accelerated intermittent theta-burst stimulation (aiTBS) for depression: a case series

**DOI:** 10.3389/fpsyt.2026.1731673

**Published:** 2026-03-13

**Authors:** Katharina Kerkel, Andreas Reissmann, Berthold Langguth, Mirja Osnabruegge, Leo Tomasevic, Karsten Litschel, Manuel Kuder, Martin Schecklmann, Stefan Schoisswohl

**Affiliations:** 1 Department of Psychiatry and Psychotherapy, University of Regensburg, Regensburg, Germany; 2 Institute of Psychology, Universität der Bundeswehr München, Neubiberg, Germany; 3 Department of Electrical Engineering, Universität der Bundeswehr München, Neubiberg, Germany

**Keywords:** accelerated intermittent theta burst stimulation (aiTBS), feasibility, home-based treatment, major depressive disorder (MDD), transcranial magnetic stimulation (TMS)

## Abstract

**Introduction:**

Transcranial magnetic stimulation (TMS) is an established treatment option for major depressive disorder (MDD) but is currently limited to clinical settings such as hospitals or doctors’ offices. Technical and logistical challenges have so far prevented its application in patients’ homes.

**Methods:**

In this pilot study, five outpatients with MDD (aged 29–65 years), living up to 120 km from a tertiary care hospital (Regensburg, Germany), received home-based TMS treatment using an accelerated intermittent theta burst stimulation protocol (aiTBS; 5 sessions per day, 1800 pulses per session). Treatment was delivered using a conventional DuoMAG XT-100 device (Deymed Diagnostics, Hronov, Czech Republic). Feasibility was evaluated through assessment of logistical challenges, device handling, safety, treatment tolerability and patient satisfaction. Depressive symptoms were measured before and after treatment using the 21-item Hamilton Depression Rating Scale (HAMD-21) and the Major Depression Inventory (MDI).

**Results:**

Home-based TMS treatment was feasible with no serious adverse events reported. A reduction in depressive symptom scores was observed. Major logistical challenges included device transport, space requirements and the need for trained personnel on site.

**Conclusion:**

Our pilot data demonstrate the feasibility of home-based TMS using a conventional clinical device, while highlighting substantial technical and logistical limitations. These limitations underscore the urgent need for the development of lightweight, portable and patient-friendly TMS devices to facilitate the delivery of neurostimulation therapies beyond clinical settings. Further studies with larger samples are warranted, using randomized controlled designs comparing home-based and clinic-based TMS to evaluate not only feasibility, but also efficacy under standardized conditions.

## Introduction

According to the World Health Organization (1), 5.7% of adults, approximately 280 million people worldwide, suffer from depression. Standard antidepressant treatments such as psychotherapy and pharmacotherapy are insufficiently effective in a substantial subgroup of patients. Since there was an urgent need for additional treatment options (2), repetitive transcranial magnetic stimulation (rTMS) was proposed as an innovative, non-invasive brain stimulation technique (3). When applied to the left dorsolateral prefrontal cortex (DLPFC), rTMS has demonstrated antidepressant efficacy in numerous randomized clinical trials and is an established evidence-based treatment option for Major Depression Disorder (MDD), particularly in case of non-response to standard therapies (3).

Recently, intermittent theta-burst stimulation (iTBS), a patterned form of rTMS delivering bursts of 50 Hz pulses repeated at theta (5 Hz) intervals, has emerged as a shorter, yet equally effective protocol (4–7). Furthermore, accelerated iTBS (aiTBS) protocols involving multiple iTBS sessions per day (up to 10) have been introduced, demonstrating similar effectiveness, good tolerability and improved cost-effectiveness (4, 8–11).

Up to now, TMS treatment remains confined to hospitals, outpatient clinics, or medical offices and is not available for administration at patients’ homes. In contrast, home-based treatment with transcranial direct current stimulation, another non-invasive neurostimulation technique, has been successfully explored in recent years (12–14).

Several aspects have so far challenged the implementation of TMS in home-based settings. First, conventional TMS devices are large, heavy, complex and expensive (15). Second, these devices are not designed for self-administration and require trained professionals to ensure precise coil placement and appropriate stimulation settings, which are critical for treatment efficacy (16). Third, safety concerns arise when treatments are administered outside clinical environments without appropriate monitoring, increasing the risk of side effects, such as epileptic seizures (17, 18).

On the other hand, limiting TMS availability to medical facilities restricts access for many patient groups, especially those with mobility or geographical barriers (19, 20). Although the first generation of portable TMS devices has been developed (21), to our knowledge, there are currently no reports on the implementation of antidepressive rTMS treatments outside clinical settings.

Given these constraints, our study aimed to explore the technical feasibility and practical challenges of delivering home-based TMS treatment in MDD patients using a conventional device following an aiTBS protocol. Specifically, we aimed to evaluate the transportability, setup, safety, treatment tolerability and patient satisfaction of the device within their home environment. This pilot study represents an initial step in assessing whether home-based TMS can be a viable treatment option despite the current limitations of large TMS devices.

## Methods and materials

All methodological procedures for the implementation of the present home-based study were approved by the ethics committee of the University of Regensburg (24-3652-101). The trial was registered at the U.S. National Institutes of Health Database (www.clinicaltrials.gov) accessible with the identifier code NCT06689592. All patients gave written informed consent prior to study participation. Due to two drop-outs (see below), seven outpatients were recruited for the home-based TMS treatment from September 2024 to February 2025. In addition to the standard inclusion and exclusion criteria for TMS treatment (e.g. previous neurological illnesses, electrical or metal implants in the head area, etc.), various logistical aspects (e.g. sufficient space at home for the TMS device and barrier-free access) were further requirements or participation in the study.

### Implementation of the home-based TMS study

**Figure 1:**
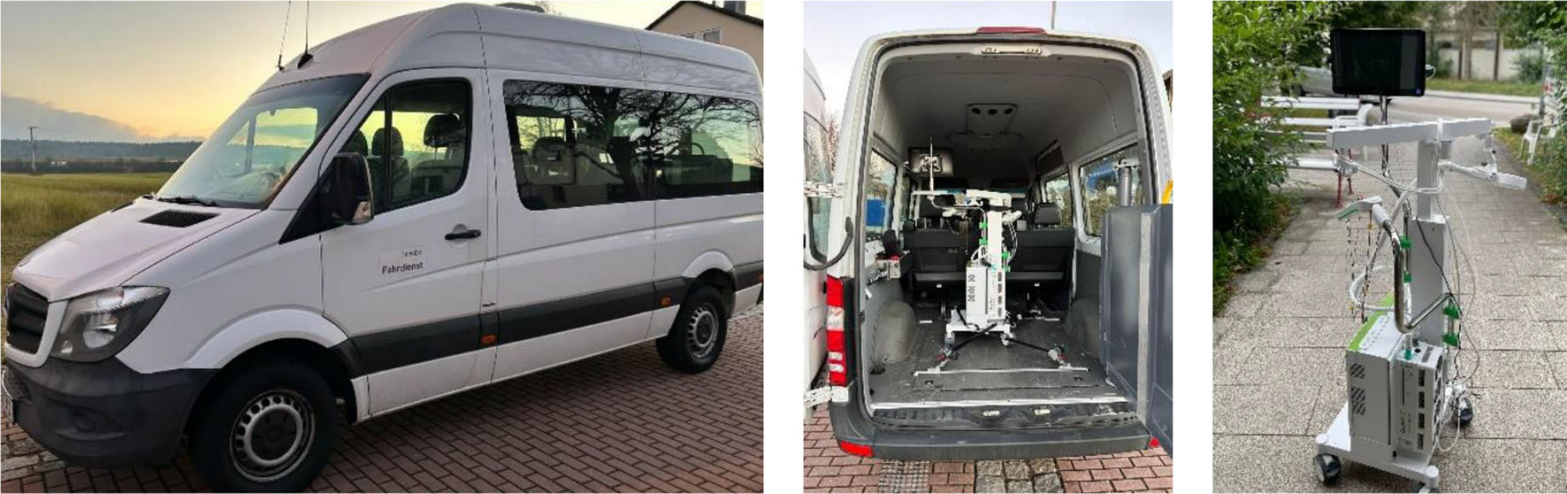
Transport of the TMS device (own source). A van of a suitable height was necessary to transport the TMS device for the home-based treatments. The TMS device was secured using straps and fixed ropes so that it could be transported safely.

Once an eligible patient agreed to receive TMS at home, an experienced psychologist and/or physician traveled to the patient’s home to conduct an initial meeting and provide detailed information about home-based TMS. On site, each patient was informed about the procedure, the purpose of the study and the fact that at least two practitioners would be at the patient’s home for the time of treatment, monitoring each session. One key point of the information provided was that an emergency physician would be called in the event of an emergency (e.g. epileptic seizure) and that treatment would be performed without a physician being immediately available. Nevertheless, all clinical staff in Regensburg are required to complete mandatory annual training in first aid and cardiopulmonary resuscitation, independent of TMS. For the present study, this ensured that, in the event of a medical emergency during a TMS session, practitioners would be able to provide immediate basic life support (e.g., placing the patient in the recovery position) until emergency medical services arrived. However, no additional emergency equipment (e.g., defibrillator, oxygen) or rescue medications were carried during home visits. In this pilot study, emergency management relied on immediately contacting an emergency physician if required. If the patient agreed to a treatment at home, a written declaration of consent was signed. Afterwards, a suitable place for the TMS device at home was identified and the necessary power supply was clarified. In a subsequent visit, the TMS device was transported to the patients’ home (see **Figure 1**).

### Transcranial magnetic stimulation

All magnetic stimulations were performed with a DuoMAG XT-100 (Deymed Diagnostics, Hronov, Czech Republic) using a figure-of-8 coil to induce biphasic pulses (default current direction, PA-AP). The DuoMAG system was chosen due to fixation of the stimulator on a mobile cart together with an integrated electromyography (EMG) device and an integrated coil holder (see **Figure 1**). For determination of the resting motor threshold (RMT), the individual motor hotspot was determined first, by administering single pulses at different positions over the left primary motor cortex.

For EMG recording, pre-gelled Ag/AgCl foam electrodes (Kendall™ H124SG, Cardinal Health) with a diameter of 24 mm were used. Prior to electrode placement, participant skin was cleaned using alcohol pads. EMG responses were recorded from the right first dorsal interosseous muscle (FDI) using a belly-tendon montage. The ground electrode was attached to the ventral side of the right forearm. Hotspot search started from a point 5cm lateral to point Cz, using a coil orientation of 45° to the sagittal midline (with the handle of the coil pointing backwards). The hotspot was defined as the skull position yielding largest and most reliable MEP responses in the first dorsal interosseous muscle (FDI) of the right hand. The subject’s motor hotspot was defined as the point where single TMS pulses evoked stable motor-evoked potentials (MEPs) with the highest amplitude. Once the subject’s motor hotspot was identified, the treatment-coil was fixated and the subject’s RMT was then measured by stimulating the individual hotspot while simultaneously recording the MEPs from the thenar muscles of the right hand using EMG (22). It was determined based on the Rossini-Rothwell method, which defines the RMT as the lowest stimulation intensity necessary to elicit MEPs of at least 50 µV in 50% of the applied pulses (23). All patients were treated with an aiTBS protocol over the left DLPFC, again, using a coil orientation of 45° to the sagittal midline. Based on the SAINT-protocol by Cole et al. (8, 9), all patients were treated with half of the SAINT-protocol using 5 stimulation sessions á 1800 pulses per day with 50 minutes interval between sessions for 5 days (overall 25 sessions). The DLPFC was localized using the adjusted Beam F-3 method (24, 25). We aimed for a target treatment intensity of 80% RMT, which was otherwise limited to 60% maximum stimulator output (MSO).

**Figure 2:**
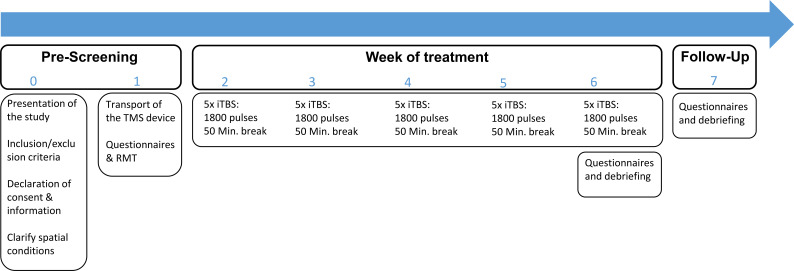
Course of the study. The figure shows the course of home-based TMS treatment for one patient. For each visit (0 - 7), the practitioners had to go to the patient. Visit 0 and 1 took place in the week before the treatment week (usually Thursday and Friday). During visit 1, the resting motor threshold (RMT) was determined by an experience practitioner. Each patient was treated 5x/day with accelerated intermittent theta-burst stimulation (aiTBS) using 1800 pulses per session with 50 minutes interval between each session (based on the SAINT-protocol by Cole et al. (8, 9). During the 50-minute breaks, the practitioners awaited the next session either at the patient’s home or outside, depending on which setting was more comfortable for the patient.

Treatments were performed with the patient seated in his/her own chair. For safety reasons, the coil was removed after each day of treatment, brought back to the clinic and reconnected the next day. A Follow-Up visit took place 4 weeks after the last treatment. **Figure 2** illustrates the course of the home-based TMS study for one patient.

### Assessment

Before the first and after the last TMS treatment session patients were interviewed with the Hamilton Depression Rating Scale (HAMD-21; 26) and completed the Major Depression Inventory (MDI; 27). In order to investigate the subjective patient experience, all patients filled out the User Experience Questionnaire after the final visit (UEQ; 28). The questionnaire is divided into six scales, which measure usability as well as user experience aspects (7-step Likert-Scale ranging from -3 to +3). The six scales include Attractiveness (Overall impression of the product), Perspicuity (Is it easy to get familiar with the product?), Efficiency (Can users solve their tasks without unnecessary effort?), Dependability (Does the user feel control of the interaction?), Stimulation (Is it exciting and motivating to use the product?) and Novelty (Is the design of the product creative?) (https://www.ueq-online.org/; access: 2025-03-21). Side effects were assessed by direct observation (e.g., visible jaw or eyebrow twitching) and by actively questioning the patient immediately after the completion of each treatment session. All reported and observed side effects were documented by one of the practitioners in a treatment protocol.

### Statistical analysis

For the course of depressive symptoms, the mean and individual sum scores of the HAMD-21 and MDI as well as the course of the sum scores (Δ score = after treatment – before treatment scores) are reported for the three measurement times: before treatment, after treatment, Follow-Up. For a better overview, the original numbering of the included patients is maintained (1 – 5). As mentioned above, potential TMS-related side effects were documented on a paper-pencil protocol and quantified by the number of patients reporting or displaying a side effect in at least one of the TMS sessions. The mean values of the UEQ were analyzed with a preset Excel sheet, which is open to the public (ueq-online.org), by Schrepp et al. (28). All statistical analyses were conducted with SPSS version 28.0 (IBM SPSS, Chicago, IL). A cost analysis was performed using both collected data and additional research on relevant economic factors.

## Results

### Feasibility

Seven patients were recruited for participation. One patient asked to stop treatment prematurely (Visit 5) due to the lack of benefit and the burden of daily TMS treatment. Another patient could not be treated at her home because of a technical failure: During transportation, the stimulator cable became disconnected from the TMS device, resulting in malfunction. This issue was not detected until after the device had already been transported back to the hospital and the patient’s treatment had commenced there. Hence, a total of five patients completed the home-based TMS treatment and were included in the present descriptive analysis.

### Efficacy

**Figure 3:**
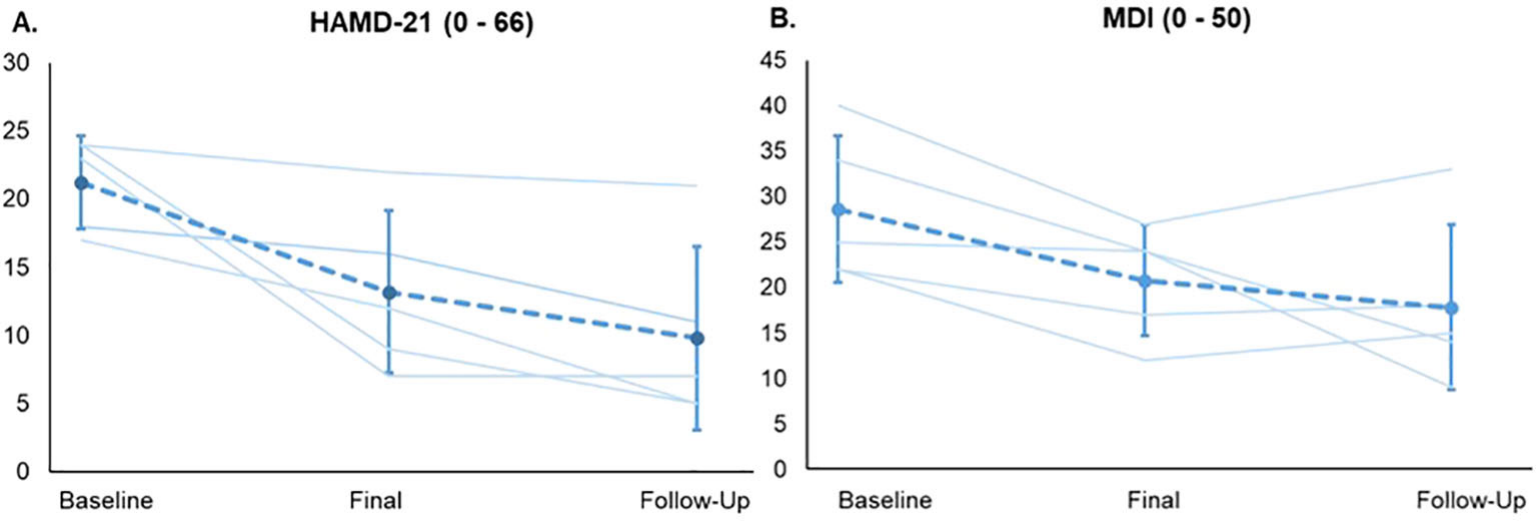
Progression of depressive symptoms. Course of the depression scores for each patient as well as the mean course for the home-based TMS study (three measurement points) for all study visits: the Hamilton depression rating scale (HAMD-21) **(A)** and the Major Depression Inventory (MDI) **(B)**. Error bars indicate SDs for the mean scores.

From before to after treatment, depressive symptoms dropped in all patients, according to both questionnaires. At the 4-week follow-up, 3 out of 5 patients were still considered in remission according to the HAMD-21, while only one patient showed a slight worsening, as assessed by the MDI. (see **Figure 3**). Demographic and clinical characteristics of the enclosed patients are provided in **Tables 1**, **2**.

**Table 1:** Demographic data per patient.

Pat.	Sex	Age	Depression diagnosis (ICD-10)	Comorbid disorders (ICD-10)	Psych. Med. (dosage)	RMT	TI	No. of Sess.
1	f	59	F33.1	-	Paroxetin (20mg)	52	42	25
2	f	65	F33.1	F42.0	Clomipramin (75mg), Venlafaxin (150mg)	54	43	25
3	f	65	F33.1	F45.41	Paroxetin (30mg)	51	41	24*
4	f	29	F34.1	F41, F68.8, F60.1	–	61	49	25
5	m	62	F33.1	-	-	57	46	25
M (SD)						55.00 (4.06)	44.20 (3.27)	24.80 (.46)

Pat., patient number; f, female; m, male; Psych. Med., psychiatric medication; M, Mean scores; SD, Standard deviation; RMT, Resting motor threshold; TI, Treatment intensity = 80% RMT; No. of Sess., Number of sessions. * Patient 3 had a doctor’s appointment during the course of treatment and, thus, missed one session. Patients were taking daily antidepressants; doses were not changed throughout the study.

**Table 2:** Depression sum scores per patient: Before and after treatment as well as Follow-Up.

	Patient	Before treatment	After treatment	After – before treatment	Follow-Up
**HAMD-21**	1	17	12	-5	5
	2	24	22	-2	21
	3	24	9	-15	5
	4	23	7	-16	7
	5	18	16	-2	11
	**M (SD)**	21.30 (4.06)	13.11 (6.27)	-8.19 (5.78)	9.80 (6.72
**MDI**	1	25	24	-1	9
	2	22	17	-5	18
	3	22	12	-10	15
	4	34	24	-10	14
	5	40	27	-13	33
	**M (SD)**	27.80 (10.35)	22.30 (11.16)	-5.50 (4.62)	17.80 (9.09)

HAMD-21, Hamilton Depression Scale: 21 items; MDI, Major Depression Inventory; M, Mean scores, SD; Standard Deviation.

### Tolerability

No serious adverse events occurred in any of the patients. The following side effects occurred: 1/5 patient reported pecking in the eye, 3/5 patients displayed lid twitching, 2/5 patients displayed jaw twitching, 1/5 patients displayed eyebrow twitching, 1/5 patients reported fatigue, 1/5 patients reported light headache, and 1/5 patients reported continuous sensations on the nose (note: some patients reported/displayed multiple side-effects).

### Patient experience: evaluation of the home-based treatment

**Figure 4:**
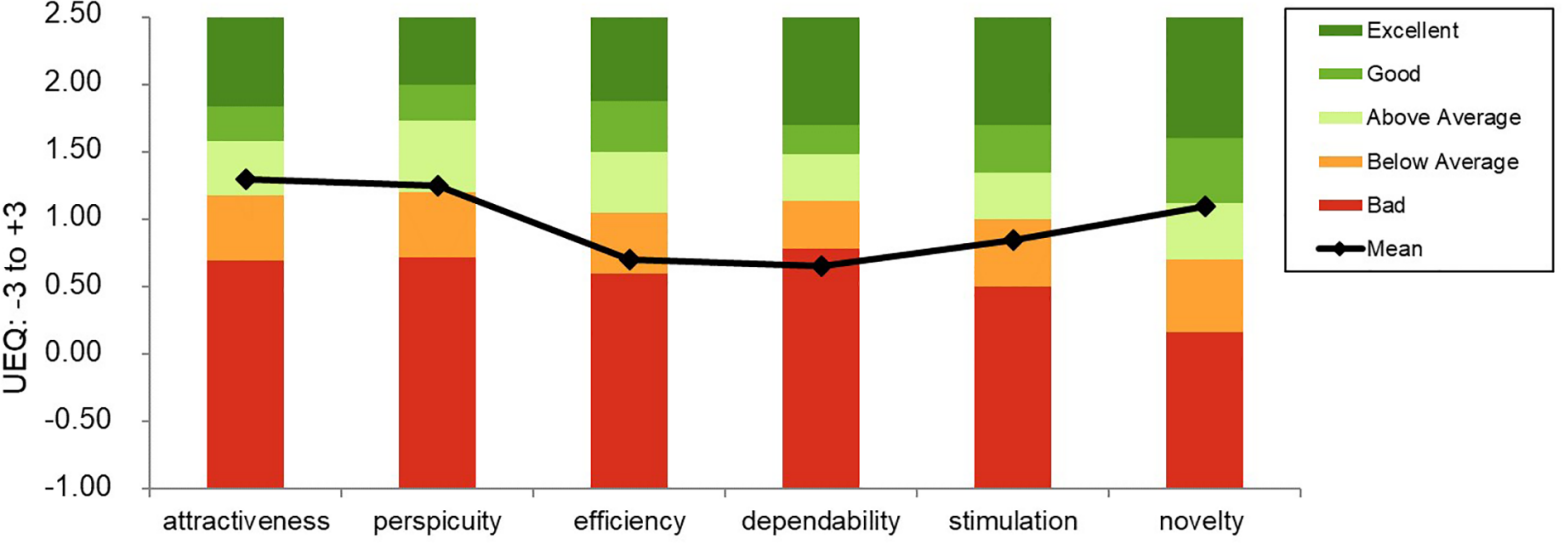
Mean evaluation of the user experience. This graph shows the mean scores of the 6 factors of the user experience questionnaire (UEQ) of all patients based on the open-source evaluation method by Schrepp et al. (28). Higher scores correspond to better evaluation (range of the transformed values: -3 (max. negative) to +3 (max. positive)).

The User Experience Questionnaire (UEQ) (28), revealed the following results: the factors *efficiency* (M = .70, *SD* = 1.15) and *stimulation* (M = .85, *SD* = 1.50) were rated “below average”. The factors *attractiveness* (M = 1.30, *SD* = 2.03)*, perspicuity* (M = 1.25, *SD* = 2.63) and *novelty* (M = 1.10, *SD* = 1.33) were rated “above average”. *Dependability* (M = .65, *SD* = 1.83) was rated “bad” (see **Figure 4**).

### Comparison of costs: clinic-based vs. home-based TMS

From a structural and economic perspective, home-based TMS showed to be more resource-intensive than clinic-based TMS. Here, we treated all patients with two practitioners being on site for the entire treatment duration (five consecutive full working days) and device transport added logistical complexity and cost. **Table 3** presents an estimated cost comparison between home-based and clinic-based TMS treatment.

**Table 3:** Estimated cost comparison between clinic-based and home-based TMS (per patient, 25 sessions).

Cost category	Clinic-based TMS (€)	Home-based TMS (€)	Source		Assumptions/ notes
Patients treated per TMS device per day (using an aiTBS protocol)	5	1 – 5			Home-based TMS: depending on the number of patients recruited for the week of treatment
Practitioners needed per treatment day	1	1 – 2			Emergencies are better handled with two practitioners in home-based TMS
Personnel time per patient (total)	5 h	40 h			Clinic: 0.2h x 25 sessions; Home-based: 8h/day x 5 days of treatment
Personnel costs	550€	1760€		29	22€/h
Room/facility costs	450€	-			Amortized clinic space
Equipment depreciation & maintenance*	600€	600€			Assumed equal across settings to avoid overestimation of cost differences despite possible lower utilization in home-based TMS
Insurance & liability	250€	300€			Higher liability for home-based treatment
Transport – vehicle rental & depreciation	–	450€			Van rental for 5 days
Transport – fuel	–	350€		30, 2025	
Travel time (personnel)	–	440€			~ 20h travel time x 22€ (Reflected in full-day personnel allocation)
Electricity	120€	14€**		Electricity prizes in Germany, 2025	Clinic vs. household rates
Opportunity costs (idle device & staff)	Low (included)	High			Clinic-based TMS allows same-day replacement of cancelled patients; home-based TMS results in a full-day loss of personnel and device availability
**Total estimated cost** **(25 sessions)**	2000 – 3000€	3900 – 4800€			Conservative estimates

*Estimated per-patient cost, calculated as the sum of device depreciation, annual maintenance and insurance/transport risk.

**Electricity costs for home-based TMS must be covered by the patient.

aiTBS: accelerated intermittent theta-burst stimulation.

All estimates are based on current market values in Regensburg, Germany (2024–2025).

Clinic-based treatment benefits from economies of scale, allowing multiple patients per device and practitioner per day.

Home-based costs assume one full-time practitioner plus assistant, local fuel prices and average household electricity rates.

## Discussion

To the best of our knowledge, this is the first study to investigate the feasibility of administering TMS for the treatment of depression entirely in patients’ homes using a conventional, non-portable TMS device. The primary aim of this study was not to assess clinical efficacy, but rather to investigate the technical and logistical feasibility of delivering home-based treatment with a standard clinical device.

Regarding the course of depressive symptoms, the antidepressant potential of an aiTBS protocol is well established in the literature and is briefly mentioned in our findings: all patients showed an improvement in depression symptoms following TMS treatment (HAMD-21, MDI). Our findings highlight substantial practical and organizational challenges regarding the feasibility of home-based TMS, particularly in terms of transporting the TMS device to patients’ homes. The device was secured only using straps and was equipped with brakes, which could have potentially disengaged during transport. Nevertheless, authorization to conduct the study was obtained from the manufacturer and a formal loan agreement was in place. Future home-based studies may mitigate the risk of damaging the device by implementing additional securing mechanisms during transport. Transporting the stimulator, which weighed approximately 20 kg and measured 1.63 m in height (including the screen of the EMG monitor, see **Figure 1**), required a high-roof vehicle and at least two trained staff members for delivery, setup and removal, as in some cases several steps had to be passed to reach patients’ homes. While one practitioner with comprehensive expertise would generally be sufficient during the actual TMS treatment, given that the risk of an epileptic seizure is considered minimal (31), the transport and setup of the device in patients’ homes would have been hardly feasibly without the support of the second practitioner. The size of the stimulator itself led to exclusion of some patients from potential study participation, since their living space was either too small or lacked appropriate access (e.g., an elevator or being on the ground floor). Further, adverse weather conditions (e.g., heavy rain or snow) posed additional risks of damage during loading and unloading. Within patients’ homes, all patients were explicitly instructed not to operate the device and to leave it undisturbed. The device was positioned to minimize interference, occasionally within a dedicated or partially restricted area. In our pilot study, there were no small children or pets present in any of the households; therefore, in households with small children or pets, future studies should only proceed with TMS treatment if the device can be secured by the adult patient in a separate room, in order to minimize the risk of accidental contact or injury. In the event of accidental damage, the study would have been discontinued and responsibility for any necessary repairs or related actions would have rested with the study team, not the patients. Generally, beyond operational safety, regulatory, liability and insurance considerations are crucial for home-based TMS. As TMS devices are certified medical devices (CE-marked), their use outside clinical environments requires compliance with applicable local regulations and institutional guidelines. Responsibility for potential adverse events, including accidental injury to patients or third parties or damage to the device, must be clearly delineated, typically resting with the clinical institution or study team. Adequate insurance coverage, encompassing device transportation, on-site operation and potential third-party liability is strongly recommended to mitigate legal and financial risks. Incorporating such measures is crucial for both participant safety and regulatory compliance in future home-based TMS implementation.

The device required access to two power outlets in close proximity. The use of household electricity further raised important concerns regarding safety, regulation and liability. Nevertheless, the fears have proven to be unfounded as the DuoMAG XT-100 operates with a universal power supply of 100–240 V AC, 50/60 Hz, allowing safe connection to standard electrical circuits outside clinical hospital environments. The device is designed according to medical electrical safety standards and includes built-in protections to ensure stable and reliable operation regardless of typical variations in external power supply conditions (32).

With respect to **tolerability**, no serious side effects occurred. In this pilot study, no additional emergency equipment or medications were available on site and emergency management relied solely on contacting an emergency physician if required. To ensure adequate safety in home-based TMS, future studies should equip staff to manage potential emergencies on site, including appropriate training (e.g., proper use of oxygen equipment) and basic emergency resources (e.g., oxygen or rescue medications such as benzodiazepines under appropriate supervision). A relative high number of minor side effects such as muscle twitches were reported. Presumable this observation was related to the home-based setting, e.g. by increased alertness to possible side effects and more communication with the patients (e.g. during treatment pauses) resulting in an increased attention on potential side effects than in a clinical setting. In terms of the relationship between the patients and the practitioners, the enhanced therapeutic relationship during home-based sessions may have contributed to the observed remission rates at Follow-Up (3 out of 5 patients according to the HAMD-21). Treatment pauses often included discussions of patients’ personal histories and depression symptom improvement. Future studies should consider controlling for such additional, potentially subconscious psychological influences arising from interactions with practitioners in order to more precisely isolate the effects of the TMS intervention itself.

Patients rated their overall experience with home-based TMS positively in terms of convenience and general impression (UEQ factors: attractiveness, perspicuity and novelty). However, the dimensions of motivation (UEQ factors: stimulation and efficiency) and trust in the device (dependability) were rated low, suggesting a certain degree of uncertainty in using complex medical technology outside clinical supervision. These findings emphasize the importance of intuitive, user-centered device design and robust safety monitoring.

Regarding structural and economic points, the costs of home-based TMS treatment were higher compared to clinic-based administration, despite identical treatment protocols. In this novel pilot study, we decided to assign two practitioners for the implementation of the study for safety reasons, resulting in substantially higher personnel costs. Moreover, transportation, setup and calibration of the TMS device in patients’ homes introduced additional logistical complexity and financial burden. To address these challenges, future studies should explore strategies to reduce on-site staffing demands, such as remote supervision or hybrid care models and determine the minimum level of oversight required to ensure safety and correct adherence to the treatment protocol.

### Home-based vs. clinic-based TMS

While home-based TMS involves substantial logistical and organizational challenges, particularly regarding device transport, setup and on-site staffing, it could still be envisioned as a complementary strategy to reduce strain on healthcare systems. In the present pilot study, each patient required the continuous presence of two practitioners across several full working days, which increased personnel costs and limited scalability compared to clinic-based administration. Transporting, setting up and calibrating the device added further logistical complexity and these organizational demands need to be considered carefully when evaluating home-based delivery.

Nevertheless, home-based TMS may offer important clinical advantages. Patients who are severely impaired or geographically distant could benefit from greater accessibility, reduced stress from travel and improved adherence due to treatment in a familiar environment. In our pilot study, all patients explicitly expressed appreciation for receiving therapy without needing to leave their homes, highlighting the potential benefits of comfort and privacy. Decentralizing TMS treatment also presents organizational opportunities. Mobile treatment models may help alleviate pressure on clinics during periods of high demand or staff shortages and could increase flexibility in the utilization of devices and personnel, particularly in rural areas, potentially reducing dropout rates.

At the same time, home-based treatment with on-site personnel remains resource-intensive, due not only to treatment time but also to travel, device transport and setup, leading to higher personnel and logistical demands. Future studies should explore strategies to reduce on-site staffing requirements, for example through hybrid care models or the use of portable TMS devices that do not require continuous supervision. Most of the logistical disadvantages observed in the present study could be avoided with smaller, more easily portable stimulators, as recently developed by Qi et al. (21). Consideration of these factors is crucial for the design of future applications and home-based device development.

### Limitations and outlook

Findings are limited to feasibility and patient experience. Practitioner experience was not formally assessed, despite its relevance for feasibility, scalability and workforce burden. These dimensions represent important areas for future research and should be addressed through dedicated qualitative or mixed-methods approaches in prospectively designed studies. Further, the present pilot study used a conventional TMS device not designed for home use. Future studies should focus on the development and validation of lightweight, portable TMS systems, ideally including automated coil positioning, remote monitoring and robust safety features. Randomized controlled trials comparing clinic- and home-based TMS protocols are needed. These should control for environmental and interpersonal variables, such as the amount of practitioner interaction or daily structure, both of which may confound treatment effects. In this context, future studies should assess expectation effects, as they may influence symptom reporting and perceived tolerability. Future studies could incorporate standardized measures, such as the Treatment Expectancy Questionnaire (TEX-Q), to better account for these potential effects. Further, this study did not systematically collect qualitative data on aspects specific to home-based TMS, such as perceived advantages and disadvantages of receiving treatment at home, sense of safety, or disruption of daily routines.

## Conclusion

This feasibility study demonstrates that antidepressive home-based TMS treatment using a conventional clinical device is technically possible and leads to symptom reduction but is associated with substantial logistical and financial challenges. This may limit its potential as a standard therapeutic approach in the future, but it could still be implemented in cases where specific needs arise. Our findings also highlight the potential for portable, autonomous TMS systems designed specifically for home-based treatment. If successfully developed, such systems could expand access to neuromodulatory therapies, improve treatment adherence and reduce the burden on clinical infrastructure, particularly for patients who would otherwise be excluded from standard care. This includes individuals with significant mobility limitations, physically vulnerable older adults, patients living in remote or underserved areas and those with intensive caregiving responsibilities that limit their ability to attend regular clinical appointments. These patients often encounter substantial logistical, physical or social challenges that may prevent consistent attendance at clinic-based sessions and may experience additional stress related to travel and disruption of daily routines. By delivering treatment directly in the home, home-based TMS can reduce travel-related burden, accommodate individual schedules and potentially improve adherence and continuity of care. Further, this approach may also be applicable in healthcare facilities without on-site TMS equipment, where a trained practitioner could deliver and supervise treatment, ensuring adherence to safety protocols. As long as a portable, autonomous TMS system has not been developed yet, maintaining appropriate monitoring and standard emergency procedures in such settings would remain essential to guarantee patient safety and broaden the clinical relevance of this method.

## Data Availability

The original contributions presented in the study are included in the article/supplementary material, further inquiries can be directed to the corresponding author.
